# An Up-To-Date Review on Biomedical Applications of Palladium Nanoparticles

**DOI:** 10.3390/nano10010066

**Published:** 2019-12-27

**Authors:** Thi Tuong Vy Phan, Thanh-Canh Huynh, Panchanathan Manivasagan, Sudip Mondal, Junghwan Oh

**Affiliations:** 1 Center for Advanced Chemistry, Institute of Research and Development, Duy Tan University, 03 Quang Trung, Hai Chau, Danang 550000, Vietnam; phanttuongvy4@duytan.edu.vn; 2 Center for Construction, Mechanics and Materials, Institute of Research and Development, Duy Tan University, 03 Quang Trung, Hai Chau, Danang 550000, Vietnam; huynhthanhcanh@duytan.edu.vn; 3 Center for Marine-Integrated Biomedical Technology, Pukyong National University, Busan 48513, Korea; manimaribtech@gmail.com (P.M.); mailsudipmondal@gmail.com (S.M.); 4 Department of Biomedical Engineering, Pukyong National University, Busan 48513, Korea

**Keywords:** palladium nanoparticles, biomedical applications, photothermal therapy, photoacoustic imaging, antimicrobial/antitumor application, gene/drug delivery, prodrug activation, biosensor

## Abstract

Palladium nanoparticles (PdNPs) have intrinsic features, such as brilliant catalytic, electronic, physical, mechanical, and optical properties, as well as diversity in shape and size. The initial researches proved that PdNPs have impressive potential for the development of novel photothermal agents, photoacoustic agents, antimicrobial/antitumor agents, gene/drug carriers, prodrug activators, and biosensors. However, very few studies have taken the benefit of the unique characteristics of PdNPs for applications in the biomedical field in comparison with other metals like gold, silver, or iron. Thus, this review aims to highlight the potential applications in the biomedical field of PdNPs. From that, the review provides the perceptual vision for the future development of PdNPs in this field.

## 1. Introduction

Metal nanostructures are gaining interest in a lot of biomedical applications owing to their shape and size; electronic, mechanic and optical properties; and their outstand resistance to corrosion and oxidation [1,2,3]. Metal nanostructures offer invaluable possibilities for new cancer therapies, targeted drug delivery, detection/diagnosis, and bioimaging [1,4,5]. Currently, gold, silver, iron, and platinum nanostructures are broadly studied in the biomedical field; several studies have taken the steps into clinical practice [6,7,8].

Besides the common unique characterizations of metals, the noble palladium nanoparticles (PdNPs) have excellent physicochemical properties, such as high thermal stability, good chemical stability, remarkable photocatalytic activity, electronic properties, optical properties, and low cost [9,10]. PdNPs can be synthesized with a wide range of sizes and shapes [9,11,12]. They can be coated with other biopolymers or molecules to create biocompatible nanoparticles with desired properties [13,14,15]. Previous reports have explored the broad applications of PdNPs in organic coupling synthesis [16,17], hydrogen storage/sensing [18], fuel cells [19], and sensors [20]. Especially, PdNPs are widely used in different applications such as a catalyst for C–C bond formation and oxidation processes in the pharmaceutical field [21,22]. However, a very small number of studies have reported the applications of PdNPs in the biomedical field. Recently, PdNPs were discovered as photothermal agents [13,15,23,24], photoacoustic agents [13,25,26], gene/drug carriers [27,28], prodrug activators [29,30], anticancer agents [31,32], and antimicrobial agents [33,34] (Table 1). To the best of our knowledge, there have been no recent and updated review articles about the application of PdNPs in the biomedical field.

PdNPs have currently attracted considerable interest in photothermal therapy (PTT) owing to their high thermal stability and optical property [13,15,23,24]. PTT is a novel therapy that uses near-infrared (NIR) laser photo-absorbers to generate heat under NIR laser irradiation. The strong absorption in the NIR region of PdNPs maximizes the penetration of light into tissue [35]. Using the optical property of material, photoacoustic imaging (PAI) has been also recently developed as a novel promising imaging technique for early tumor diagnosis and management [36,37]. To enhance the photoacoustic signal from the tissue, photoacoustic agents are often required [38]. With the intrinsic optical property, PdNPs have been very recently studied as photoacoustic agents; the obtained results show the promising potential of PdNPs as an effective photoacoustic agent for PAI [13,25,26]. Another major application of PdNPs in biomedicine is gene/drug delivery [27,28]. The high surface area for loading the high amount of drugs, stability, and nontoxicity of PdNPs makes them work as efficient nanocarriers for gene/drug or other small molecules [27,28]. With their outstanding photocatalytic properties, PdNPs have been discovered as prodrug activators [29,30]. This application was well studied on 5-fluorouracil (5FU) and gemcitabine [29,30]. The antitumor mechanism of PdNPs was recently exploited and the anticancer property of PdNPs was initially tested on several cancer cell lines. The effectiveness of PdNPs as promising antimicrobial agents was tested on several bacteria strains [33,34]. 

We are in the early stage of the discovery journey towards the realistic biomedical application of PdNPs. There is a long way to go to transfer the PdNPs-based nanotechnology to clinical applications. To ensure the successful translation, we need a comprehensive understanding of the physicochemical properties, effects in vitro and in vivo, toxicity, bio-distribution, pharmacokinetics, and pharmacodynamics of PdNPs. In 2015, Dumas et al. published the first minireview reporting the emergence of PdNPs in nanomedical fields [23]. At the time, a few applications on the prodrug activation, photothermal therapy, and anti-cancer/anti-microbial therapy applications was discovered and reported in that minireview. This review has been motivated to share up-to-date information on the potential applications of PdNPs in the biomedical field. At first, the synthesis strategies, stabilization procedures, and physical properties are briefly presented for the design of PdNPs toward biomedical applications. Next, the most recent works on the applications of PdNPs for cancer/infection PTT, photoacoustic imaging, antibacterial application, antitumor therapy, gene and drug delivery, prodrug activator, biosensor, and multifunctional nanoparticles are extensively reviewed. The overview of toxicity, pharmacokinetic, and biodistribution of PdNPs was introduced. In the end, a perceptual vision is given for the future biomedical development of PdNPs.

## 2. Synthesis and Characterization of Palladium Nanoparticles for Biomedical Applications

### 2.1. Synthesis

The preparation of PdNPs can be done via chemical, physical, or biogenic routes. Among these methods, the chemical and biogenic methods are commonly used to synthesis the PdNPs for biomedical applications. The size, morphologies, dimensions, and surface capping agents of PdNPs strongly impact their characterizations. The emergence of PdNPs for biomedical applications requires their safety or toxicity as well as dispersion stability in the biological environment. Therefore, new synthetic methods to optimize these futures are being developed.

The chemical route allows for the adjustment of reaction conditions, such as the concentration of precursors, additives or surfactants, temperature, and pH, to achieve the desired nanomaterial [52]. For example, porous PdNPs and palladium (Pd) nanocubes were synthesized by a similar procedure that used H_2_PdCl_4_ as a precursor, sodium borohydride (NaBH_4_) as a reducing agent, and cetyl trimethylammonium bromide (CTAB) as a stabilizer agent; however, the time and temperature were controlled to form the PdNPs with different shapes [53,54]. Tang et al. prepared the Pd nanosheet using Pd(II) acetylacetonate (Pd(acac)_2_) as a precursor, poly(vinylpyrrolidone) (PVP) as stabilizer, and sodium bromide (NaBr) as a reducing agent [40]. The using of toxic reducing agents (NaBH_4_ or NaBr) and less biocompatible stabilizers (i.e., CTAB) is the big disadvantage of the chemical approach. Plasma synthesis methods such as atmospheric pressure (AP) alcohol cold plasma [55] and dielectric barrier discharge plasma [56] have been recently developed for the reparation of PdNPs and other metal nanoparticles. The AP alcohol plasma is a simple, green, and efficient method to synthesis the small size metal nanoparticles with tunable crystallization and the crystal face [55]. The dielectric barrier discharge plasma is a very efficient and rapid process to produce the very fine and surfactant-free nanoparticles [56]. With the benefits of the plasma synthesis methods, more comprehensive studies need to be conducted to apply these methods in biomedical-oriented PdNPs preparation.

The biogenic route offers some advantages to biomedical applications of PdNPs, including the absence of toxic solvents, toxic reducing agents, and toxic stabilizers. PdNPs can be synthesized by plant-extracts, bacteria, alga, fungi, viruses, etc. The resulted PdNPs from biogenic methods have good biocompatibility and well-dispersed stability. The utilization of plant-extracts to synthesis biocompatible metal nanoparticles is a common method. The components of plant-extracts play a dual role as a reducing agent and a stabilizer. Particularly, PdNPs, which were synthesized by white tea (*Camellia sinensis*) extract, have a spherical shape with the size from 6 to 8 nm and contained phenols and flavonoids. They were applied for antioxidant, antibacterial, and antiproliferative activates on the human leukemia cell line [31]. PdNPs, which were synthesized by *Diospyros kaki* leaves, were in sizes ranging from 50 to 120 nm and showed strong antibacterial efficacy on *Escherichia coli (E. coli)* and *Staphylococcus aureus (S. aureus)* [46]. Porous PdNPs with rough protuberances were prepared a chaga mushroom *(Inonotus obliquus)* extract [27]. These nanoparticles retained the anticancer effect of the chaga mushroom and had an absorption peak in the NIR reagent. Thus, they can be applied for anticancer therapies.

The size of PdNPs may be controlled more easily by the chemical routes than the biogenic routes. By adjusting the temperature, time duration, or the precursor and reducing agent concentration of the chemical reaction, PdNPs with a wide range of sizes can be obtained. For instance, the size of monodispersed PdNPs can be accurately achieved from 5 to 10 nm by controlling the growing time [57]. In another work, the size of mesoporous PdNPs was controlled from 25 nm to 42 nm by the use of cationic surfactants and triblock copolymers [58]. Recently, our group developed a green and simple method to prepare porous flower-shaped PdNPs with controlled sizes [25]. The size of these nanoparticles can be resulted from 25 to 150 nm by adjusting the concentration of the chitosan polymer, which worked as a directing agent and a size-controlling agent.

### 2.2. Functionalization Procedures

Surface functionalization is an important step to control the interactions of nanoparticles with the body. Surface functionalization can be purposely fabricated to provide the targeting ligand for specific binding of nanoparticles on the cells, for enhancing the drug loading amount, for enhancing the cellular internalization ability of nanoparticles, or for hiding nanoparticles from the immune system. The surface of nanoparticles can be functionalized with small molecules, polymers, or biomolecules [59]. For example, the ultra-small Pd-nanosheets (SPNS) were functionalized using polyethyleneimine (PEI) to enhance the uptake by cells [40]. Our work used the targeting cancer cell RGD peptide to modify the surface of chitosan oligosaccharide-coated PdNPs [13]. Both in vitro and in vivo experiments showed the high accumulation of target ligand-modified PdNPs on the cells and tumor [13,40]. To enhance the drug loading capacity, PdNPs were functionalized with PEG-hydrazine to provide the hydrazine bond for the cancer drug doxorubicin (Dox) binding [50]. To prolong the circulation half-life of Pd nanosheets, the small ligands, such as glutathione (GSH), were introduced on the surface of nanoparticles [40].

### 2.3. Stabilization Procedures

The long-term stability of dispersion nanoparticles is an important issue for biomedical applications. Stabilization of PdNPs typically can be achieved by introducing the ligand (i.e., organic ligands, surfactants, or polymers) on its surface. The electrostatic or steric interactions between nanoparticles, which were created by the coating ligands, are the key for stabilizing the metal nanoparticles. The protection of PdNPs can be also gained by supporting them on the inorganic materials [60].

The adding of organic ligands is the most-used method for stabilizing PdNPs. The strong binding of organic ligands on the surface of PdNPs prevents the aggregation of the nanoparticles. Among organic ligands, sulfur-based ligands, phosphorus-based ligands, and nitrogen-based ligands are commonly used [9]. For example, poly(ethylene glycol) monomethyl ether thiol (mPEG-SH) was used to replace the cetyltrimethylammonium chloride (CTAC, a toxic shape control agent) on the surface of PdNPs to enhance the stabilization and biocompatibility of PdNPs [24].

The surfactants are often used for stabilizing the metal nanoparticles. For example, Poloxamer 407 is a surfactant with high proportions of polyoxymethylene; thus, it was used to stabilize the graphene oxide-PdNPs [61]. The hydrophilic chains of Poloxamer 407 strongly assisted the uniform dispersion of these nanoparticles.

The polymers, especially biopolymers, are introduced to prevent the agglomeration of PdNPs. Furthermore, biopolymers are also able to enhance the biocompatibility of PdNPs. In particular, the poly (vinylpyrrolidone) (PVP), which is a commercially available and low-cost polymer, was used to stabilize small palladium nanosheets (SPNS) [40]. In other work, polypyrrole was used to coat flower-like PdNPs to improve the stability as well as the heat conversion efficiency for cancer PTT [15]. The PVP-stabilized SPNS gave a high efficacy of near-infrared (NIR) PTT [40]. Chitosan (CS) and chitosan oligosaccharide (COS), which are marine-derived biopolymers, were also implemented to stabilize the PdNPs. Owing to the highly positive charges and biocompatibility of CS and COS, the CS-coated PdNPs and COS-coated PdNPs have stabilized well in an aqueous medium and have excellent biocompatibility [13,25].

The supporting of PdNPs on the most widely used inorganic matrices, such as silica, titanium dioxide, and alumina, to form nanoreactors, is an important strategy to protect the PdNPs, especially for the catalytic and photocatalytic applications [60]. The use of these nanoreactors can improve the dispersion and the recyclability of PdNPs on C–C coupling reactions [60]. Balbín et al. prepared the PdNPs on two different mesoporous silica supports for effective catalysts and cytotoxic agents against cancer cells [48]. The results proved that the hybrids with a lower load content of PdNPs, which have a better distribution of nanoparticles, were the better effective catalysts. Further, the hybrid materials also have good recyclability.

### 2.4. Physicochemical Properties

PdNPs can be synthesized in numerous shapes (e.g., sphere, cubes, flower, octahedrons, rods, etc.) owing to the face-centered cubic (FCC) crystal structure [62,63]. For example, the formation of Pd octahedrons, Pd cuboctahedrons, or Pd cubes is contingent on the ratio of the growth rates along the (111) and (100) directions [64].

Nanoparticle size plays a key role in its stability, blood circulation half-life, biodistribution, and elimination. PdNPs with the desired size can be obtained through different synthesis approaches and adjustment of different parameters for different applications. The smaller PdNPs have better antibacterial properties in comparison with the bigger ones [33]. The smaller PdNPs also have longer blood circulation half-life than the bigger one [65].

The localized surface plasmon (LSPR) properties of PdNPs expand their applications to photoacoustic imaging, photothermal therapy, and biosensors. Plasmonic Pd nanospheres have a higher susceptibility to refractive index changes than gold (Au) nanospheres and silver (Ag) nanospheres [66].

## 3. Palladium Nanoparticles for Cancer/Infection Photothermal Therapy

Photothermal therapy (PTT) is gaining great attention due to its potential in the treatment of cancer and infection [23,67,68,69]. This therapy employs the photo-absorbers that convert the photon energy to heat for the thermal ablation of cancer cells and bacteria [67]. The use of external laser irradiation with controllable dosage and diameter of the laser beam, allows PTT to be a highly selective and noninvasive therapy [70]. PTT can eradicate the primary tumor or metastasis tumor of many cancer cell types. Due to the advantages of PTT, many research groups have focused on developing the new advanced nanomaterials for PTT. Among them, the metal nanostructures are promising candidates for photo-absorbers owing to their localized surface plasmon resonance [71].

The ideal photothermal agent should include the important properties: water dispersion, small size, uniform in shape, high absorption in near-infrared (NIR) region, high photostability, high biocompatibility, targeting to the cancer cell/bacteria, and easiness to clean from renal [72,73]. The photo-absorbers were required to have strong absorption in the NIR region (700–2500 nm), which is an “optical window” with the lowest light scattering and absorption of tissue; so, the absorbed light can penetrate deeply into the tissue [72,74].

Currently, PdNPs are emerging as an efficiency photothermal agent for PTT due to their high photothermal conversion efficiency, photostability, diversity in shape, size, and high absorptions in the NIR region. As reported by Huang et al., the ultrathin hexagonal Pd nanosheets with tunable absorption peaks in the NIR region showed efficient photothermal conversion, high biocompatibility, and better photostability when compared to gold or silver nanostructures [39]. The temperature of the 27 ppm Pd nanosheet solution rose from 28.0 °C to 48.7 °C after 10 min of irradiation (808 nm, 1 W); meanwhile, the temperature of the control solution increased by only 0.5 °C [39]. Tang et al. modified the small Pd nanosheets (SPNS) with reduced glutathione (GSH) [40] to improve the prolonged blood circulation of nanoparticles, which facilitates the accumulation of nanomaterials in the tumor via the enhanced permeability and retention (EPR) effect. The temperature of the solution containing 30 ppm SPNS rose from 17.0 °C to 52.3 °C after 10 min of irradiation (808 nm, 1 W). To improve the targeting tumor accumulation, the SPNS was modified by polyethyleneimine (PEI). The in vivo experiment on tumor-bearing mice achieved good results with the tumor elimination completely. Interestingly, the GSH-Pd nanosheet can be easily cleared from the body through the renal excretion route, going into the urine. Xiao et al. have also recently reported small size porous PdNPs, which are attractive photo-absorbers due to a high photothermal conversion efficiency (93.4%) [24]. In 2018, our group developed chitosan oligosaccharide-coated PdNPs, which was functionalized with the RGD peptide for PTT and PAI for the first time [13]. The chitosan oligosaccharide enhanced the biocompatibility of PdNPs and the RGD peptide improved the nanoparticle accumulation in MDA-MB-231 breast cancer cells; thus, we achieved the good results on an in vivo experiment using a tumor-bearing mice model with intravenous injection. After 20 days, the thermal destructed tumor was reduced and completely healed without any adverse side effects.

Multi-antibiotic resistance becomes a big problem in modern medicine. Thus, the design of novel therapy for the treatment of infection is crucial. In 2019, our group developed the photothermal responsive membrane for the effective treatment of infected wounds [41]. Due to the good photothermal behavior of PdNPs, they were chosen as a photothermal agent to embed into the photothermal responsive chitosan/polyvinyl alcohol membrane. The developed membrane exhibited excellent biocompatibility, high porosity, a high degree of swelling, high moisture retention, and high photothermal performance. The in vitro test further demonstrated that the developed membranes able to kill *E. coli* bacteria under the 808 nm laser irradiation.

With their biocompatibility, photostability, high absorption in the NIR region, high photothermal conversion efficiency, diversion in size and shape, as well as cost-effectiveness, PdNPs are promising candidates for cancer/infection photothermal therapy.

## 4. Palladium Nanoparticles for Photoacoustic Imaging

In recent years, molecular imaging techniques have become important tools for earlier detection, more accurate diagnosis, and improving serious disease management and treatment [37]. In that, PAI has been recently developed as a novel promising imaging technique for early cancer diagnosis [36]. PAI uses the external light to excite the target and detect the generated sound waves to form the ultrasound image [36,75]. With non-ionization, portable, and profitable ultrasound imaging devices, PAI is suitable for real-time imaging and long-term monitoring of disease progression or therapeutic effect [76].

To enhance the contrast of PAI, exogenous photoacoustic (PA) agents like nanoparticles are often used. The PA agents, which have absorption in the NIR range, are commonly utilized in PAI due to its long penetration depth in biological samples. A stable and efficient contrast is highly desirable for PAI. Gold nanostructures are commonly studied as a PA agent for PAI. However, some reports demonstrated that the structures of the gold nanoparticles are demolished after long-time laser irradiation [26]. For example, the photothermal stability test showed that the structure of gold nanorods destroyed easily and aggregated quickly after 5 min laser irradiation at a power density of 80 mW/cm^2^ [26,77].

The potential of PdNPs as PA agents was not studied until Nie et al. reported on a Pd nanosheets (PdSs)-based PAI system [26], displaying a highly stable and effective acoustic signal. The acquired PA signal from the tumor was 3.5 times higher than the initial signal and reached a stable level after 24 h; meanwhile, the control group (phosphate-buffer saline) showed a weak signal for 24 h. The cytotoxicity study of PdSs in a mouse model evidenced that the major organs of the treated mice have no noticeable damages. The proposed PdSs-based PAI is very promising for earlier detection of tumors. In 2018, our group reported chitosan oligosaccharide-coated PdNPs, which were functionalized with the targeting cancer cell RGD peptide (Pd@COS-RGD) as effective PA agents [13]. We acquired the tumor imaging of mice after the tail vein injection with one dose of Pd@COS-RGD by the PAI system. The high accumulation of Pd@COS-RGD in the tumor region facilitated the production of a clear image of the tumor tissue by the PAI system. With active targeting ability, PdNPs is an excellent contrast agent of PAI.

PdNPs can be combined with other materials to create multimodal imaging for highly advanced imaging techniques. For example, Pd nanoplates were coated with gold to create the core-shell Pd@Au nanoplates (a diameter of about 15 nm and a thickness of 1.8 nm) for the PAI and computed tomography (CT) imaging applications [42]. The PA imaging and CT imaging after 24 h injection of Pd@Au nanoplates showed clearly the tumor of the mice. The Pd@Au nanoplates have the potential for both PA imaging and CT imaging in early cancer detection and management.

## 5. Palladium Nanoparticles for Antibacterial Therapy

**Figure 1:**
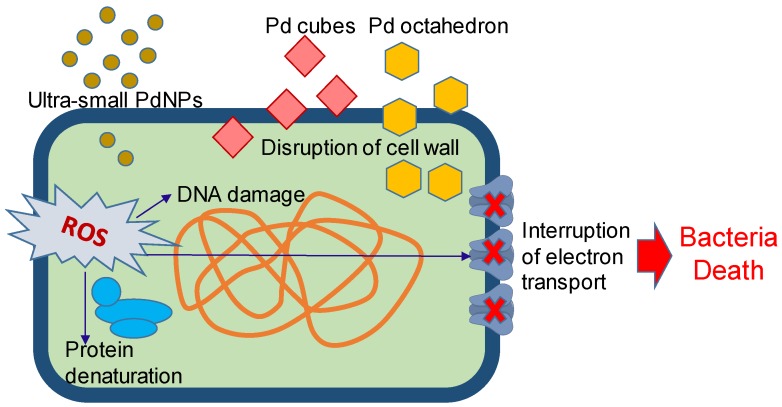
Illustration of the antibacterial mechanism of palladium nanoparticles (PdNPs). The facet of a Pd nanocrystal is able to disrupt the membranes of microorganisms. PdNPs are capable to generate the reactive oxygen species (ROS), which cause cell membrane damage, DNA damage, protein denaturation, and interruption of electron transport, resulting in the death of bacteria.

The antibacterial property of PdNPs was recently discovered and the reported data showed that PdNPs have a high potential for antimicrobial applications [31,33,46,47,78,79,80]. As illustrated in Figure 1, the antibacterial mechanism of PdNPs included both physical and chemical modes. The facet of a Pd nanocrystal is able to disrupt the membranes of microorganisms [34]. PdNPs are capable to generate the reactive oxygen species (ROS), which cause cell membrane damage, DNA damage, protein denaturation, and interruption of electron transport, resulting in the death of bacteria [34,79]. The experimental data proved that the anti-pathogenic activity of PdNPs is better than that of Pd^2+^ ions [33].

The antimicrobial effect of PdNPs strongly is dependent on their size and shape. The ultra-small PdNPs with a difference of 1 nm in size showed that the smaller PdNPs were more toxic to *E. coli* than the large ones, and the ultra-small PdNPs showed a very high antimicrobial effect at even very low concentrations (10^−9^ M) [33]. The Pd nanocrystals with two different shapes (i.e., Pd cubes and Pd octahedron) showed distinct antibacterial activity on Gram-positive and Gram-negative bacteria [34]. The facet-dependent oxidase and peroxidase-like activities of Pd nanocrystal help them have excellent antibacterial properties by the generation of reactive oxygen species (ROS). To Gram-positive bacteria, the faceted Pd cubes have a more effective killing ability than faceted Pd octahedrons; meanwhile, the octahedrons can penetrate into Gram-negative bacterial membranes in a higher number than Pd nanocubes, thus resulting in higher antibacterial activity [34].

PdNPs, which were synthesized by biogenic methods, also showed a good antibacterial property. For example, PdNPs, that were synthesized using biomass waste from petals of *Moringa oleifera* as a natural reducing and capping agent, showed excellent antibacterial activity against *Enterococcus faecalis* [32]. Or, PdNPs, which were synthesized by a green method using white tea extract (named Pd@W.tea NPs), also exhibited antibacterial activity [31]. The experimental results (on *Staphylococcus epidermidis* and *E. coli*) showed that Pd@W.tea NPs have a better antibacterial property than white tea extract, possibly due to the strong interaction of Pd@W.tea NPs to the bacteria.

The combination of PdNPs with other metals might create a new composite with high antibacterial properties. For example, the incorporation of PdNPs and silver nanoparticles (AgNPs) significantly improve the bactericidal acidity of the developed composites (hydroxyapatite/chitosan/Ag@Pd), and these scaffolds can be used for dental surgeries [81].

## 6. Palladium Nanoparticles for Antitumor Therapy

**Figure 2:**
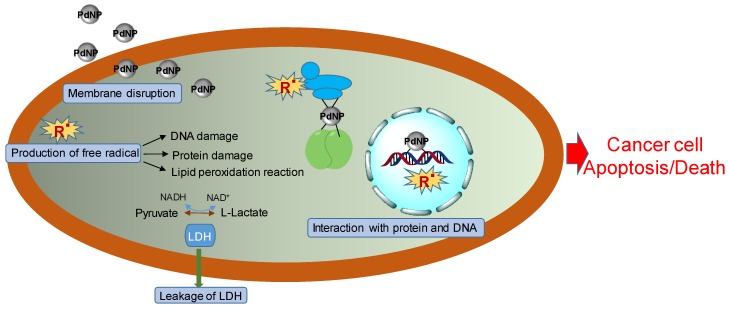
Illustration of the antitumor mechanism of PdNPs. The antitumor mechanism of PdNPs includes the physicochemical interaction of PdNPs with the functional groups of proteins, with nitrogen bases, phosphate groups of DNA, the generation of free radicals, the leakage of lactate dehydrogenase (LDH), and the disturbance of the cell cycle.

Besides the antibacterial property, PdNPs also have toxicity to the cancer cell line. As shown in Figure 2, the antitumor mechanism of PdNPs includes the physicochemical interactions of PdNPs with the functional groups of proteins, nitrogen bases, phosphate groups of DNA [82], the leakage of lactate dehydrogenase (LDH) [83], the generation of free radicals [84], and the disturbance of the cell cycle [85]. Proteins and DNA might transfer to inactive states by interaction with PdNPs. LDH plays an important role in the production of usable energy for cells. The LDH levels can be increased in many cancer cell types [86], and the leakage of LDH causes a serious problem to all functions of cancer cells. The generation of free radicals, which includes both the reactive oxygen species (ROS) and reactive nitrogen species (RNS), causes the DNA damage, protein damage, and lipid peroxidation reaction [87].

To date, very few studies have discovered the antitumor effect of PdNPs. In 2014, Balbín et al. reported the first evaluation of the anticancer activity of PdNPs in human cancer cells [48]. PdNPs were supported on the mesoporous silica-based materials and their cytotoxicity was tested on five different human cancer cell lines. The mesoporous silica does not have cytotoxic activity; thus, PdNPs play the main role in the anticancer effect of hybrid materials. The in vitro results showed that the inhibition of cancer cell growth of hybrid materials has quality-dependent, dose-dependent, and cell line-dependent properties. Pd@W.tea NPs had both the antibacterial effect and the anticancer effect [31]. The induction of apoptosis and G2/M cell-cycle disturbances on leukemia cells proved the anticancer cell effects of Pd@W.tea NPs [31]. PdNPs, which were synthesized using biomass waste from petals of *Moringa oleifera*, functioned as naturally reducing and biocapping agents. The MTT test on human lung cancer carcinoma cells (A549) and peripheral lymphocytes normal cells displayed the potential for further anticancer studies via tumor cell lines [32]. The PdNPs, which were synthesized using a plant extract (*Bauhinia variegate*), exhibited potent anti-proliferative efficacy against MCF-7 cells with a low IC50 value of 41.37 µg/mL [49].

## 7. Palladium Nanoparticles for Gene and Drug Delivery

To overcome the inherent limitations of conventional drug therapies with many limitations, such as low selectively, rapid excretion, and severe toxicity, the controlled drug delivery system has been gaining attention [88,89]. The nanoparticles can be designed to carry drugs and genes with a targeting ability and a controllable rate of molecule release. Drug molecules can be directly loaded onto the pore of the nanoparticles or indirectly conjugated with the nanoparticles by a linking molecule.

The high porosity of PdNPs was the genius property for loading genes and drugs. The fluorescein-labeled thiolated DNAzymes (FAM-Dz), which were designed to silence the HCV *NS3* gene, were loaded into the porous gold nanoparticles (AuNPs), porous platinum nanoparticles (PtNPs), and porous PdNPs; then, the loading/release profiles of FAM-Dz among those nanoparticles were examined. Comparing porous AuNPs to porous PtNPs, porous PdNPs showed better performance in gene loading and releasing [28]. For another example, the porous PdNPs which was synthesized with the aqueous extract of chaga mushroom could carry the cancer drug Dox by the electrostatic interaction between Dox and the absorption molecules on the nanoparticles’ surface [27]. The Dox was fully loaded after 6 h of incubation under ambient conditions. The acid pH (5.6) environment of the tumor tissue or cell lysozyme could allow >92% drug release that is much higher than the neutral environment (about 30%) [27]. The therapeutic drug can be also indirectly conjugated to the PdNPs through a linking molecule. For example, Dox, which was glued to PEGylated PdNPs through a hydrazine bond, showed a pH-responsive releasing profile in human cervical cancer cells (HeLa) and strong anti-tumor efficacy against HeLa tumor xenograft models in vivo [50].

As discussed in Section 6, PdNPs have anticancer activity on many cancer cell lines, resulting in potential toxicity, which may affect the results of drug-delivery studies. The cancer drug effects on targeted cells/tumors need to be carefully evaluated the cytotoxic of mono-modal PdNPs and that of dual-modal cancer drug-loaded PdNPs. The in vitro experiment revealed that 20 µg/mL of PdNPs, which was synthesized by chaga mushroom extract, was able to kill approximately 20% of HeLa cells; meanwhile, 30% of HeLa cells were killed by the Dox-loaded PdNPs (20 µg/mL of Pd) [27]. Nonetheless, this issue was not considered in some reports. For example, the effect of PdNPs alone on HeLa cells was not tested in Reference [50].

To ensure the successful delivery of gene/drug to targeting tissues, the concentration of PdNPs at which PdNPs do not cause the toxicity to the non-targeted tissue needs to be examined. The in vitro cell viability assay revealed that porous PdNPs with a concentration of up to 0.5 eq. exhibited ignorable cytotoxicity to NS3-Huh7 cells (human hepatocarcinoma cells transfected with hepatitis C virus nonstructural protein 3 replicon) [28]. Thus, the concentration of PdNPs at 0.5 eq. can be used for gene/drug delivery studies.

## 8. Palladium Nanoparticles for Prodrug Activation and Transformation Processes

Biorthogonal reactions allow the formation or cleavage of chemical bonds at a specific position of medical molecules in the physiological environment. These reactions are beneficial for the labeling, tracking, activation, transformation, and manipulation of medical molecules. 

Biorthogonal reactions can be applied to activate the nontoxic prodrug to the toxic drug for reducing side effects of cancer treatment. Based on the unique catalytic properties and biocompatibility of PdNPs, the Pd-mediated catalysis reaction could enable the activation of inactive prodrugs. The cancer drug 5-fluorouracil (5FU) developed about 50 years ago but the toxicity has limited its clinical efficacy [90]. Recently, the heterogeneous Pd^0^, which was able to catalyze the nontoxic precursor to 5FU at the extracellular level, was developed to reduce the systematic toxicity of 5FU [30]. The heterogeneous Pd^0^ catalyzed a biologically inert precursor to 5FU via functionalization on its N1 position [30]. As another example, the heterogeneous Pd^0^ catalyzed biologically inert precursors to cytotoxic gemcitabine [29]. The precursors of gemcitabine were introduced into the Pd^0^-cleavable groups in positions that mechanistically respond to gemcitabine’s pharmacological activity [29]. The introduction of Pd^0^-cleavable groups reduced the drug’s cytotoxic activity more than 23 times, as demonstrated by cell viability studies in pancreatic cancer cells [29]. The in vivo local catalytic activity of Pd^0^-resin in zebrafish yolk showed its potential in spatially controlled activation of potentially toxic compounds.

Pd-mediated transformations have been successfully applied in a series of biological reactions. For instance, Pd functionalized fluorescent microspheres have been developed for the in-cell dual-drug synthesis [91]. With the cyclic-RGD cancer targeting functionality, these catalysts can perform multiple biorthogonal transformations in vivo [91]. Due to the aggregation and/or passivation of PdNPs in biological media, the Pd-based nanocatalytic constructions need to be developed. The mesoporous silica shell covered-PdNPs have been developed for Pd-catalyzed depropargylations and Suzuki–Miyaura intermolecular couplings in bio-relevant aqueous media [92]. The management of chemical reactions in living systems is a difficult issue. To mimic the allosteric regulation mechanism of bi-enzymes, the light-controlled bio-orthogonal catalyst was developed [93]. Through the light-induced structure change, the catalytic reaction can be regulated. The novel versatile and adaptable Pd-based bio-orthogonal catalysts can be employed to a number of chemical reactions in living cells for high precision imaging and therapy.

## 9. Palladium Nanoparticles for Biosensor

Biosensors are devices using the high specificity of biological reactions to detect target analytes. The first element of a biosensor is the bioreceptors, which provide the binding of a specific analyte to the sensor and the second element is the physical transducer that takes responsibility for translating the bio-reaction into a measurable impact (i.e., electrical signal, optical emission, or mechanical motion) [94].

Dopamine (DA) is an important catecholamine neurotransmitter and the detection of DA is critical for the early diagnosis and prevention of some diseases, such as Parkinson, Alzheimer, and schizophrenia. Owing to the admirable electrocatalytic for DA, Yi et al. inserted PdNPs into nanoporous gold (NPG) to fabricate the DA biosensor [51]. The combining effects of the electrocatalysis of Pd for DA with the 3D self-supporting bi-continuous nanoporous structure of the NPG wire greatly enhanced the response current as well as the electrochemical signal. With high sensitivity, broad detection range, and excellent selectivity, this sensor is very promising for the detection of traced DA.

To demonstrate that nanostructuring can improve the performance of biosensors, Soleymani et al. designed a device architecture that consists of a wide range of nanostructured elements to perform the response of many different sensors parallel on a single chip for the same sample [95]. Using metal electrodeposition under a wide range of plating conditions, a variety of differently nanostructured Pd electrodes in the apertures was generated to complete the microelectrode array. The strong binding affinity of Pd for thiols enabled the functionalization and attachment of biomolecular probes on the nanostructured microelectrodes. The excellent performance of nanoscale biosensors has great promise for the development of high-performance diagnostic tools for biology and medicine.

## 10. Multifunctional Palladium Nanoparticles

**Figure 3:**
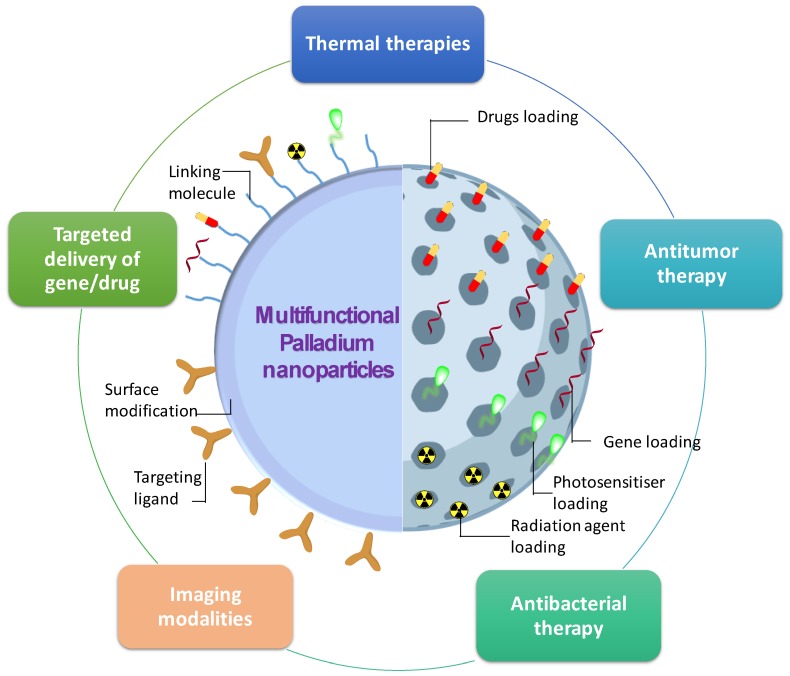
Illustration of the multifunctional PdNPs for multi-applications.

PdNPs have the potential to combine various functionalities to form the multifunctional PdNPs through nanostructure synthesis, molecules loading, and post-surface modification (i.e., coating and functionalization processing). As illustrated in Figure 3, the multifunctional PdNPs can be applied for thermal therapies, different imaging modalities, antibacterial therapy, antitumor therapy, and targeted delivery of gene/drug. With multi applications, PdNPs are able to enhance their abilities on the biomedical diagnosis and therapies with minimum side effects.

PAI and PTT are based on the principle of using the external laser to acquire tumor/tissue images and ablation of the tumor, respectively. Taking advantage of using the laser irradiation with the same wavelength, the PdNPs can be used for PAI-guided PTT. For example, the Pd@COS-RGD was developed to be used as a nano-theranostic agent for PAI-guided PTT [13]. Thanks to the high accumulation of Pd@COS-RGD in the tumor and its strong NIR absorption, the clear imaging of the tumor was able to be acquired by the PAI system. The PAI images help the photothermal system localize the location of the tumor accurately; thereby, the laser irradiation can eliminate the tumor completely and minimize the harmfulness to the surrounding healthy tissue.

To a large tumor, only PTT may not completely eradicate the whole tumor. To improve the tumor ablation efficiency, the dual effects of chemotherapy (CMT) and PTT can be combined into a single PdNPs. Fang et al. reported the cancer drug Dox loaded onto a Pd nanosheet covered the hollow mesoporous nanoparticles for the combined chemo-photothermal therapies [43]. Besides the low pH of the cancer cell factor, the generated heat by NIR irradiation on the Pd nanosheet shell also induced the fast release of Dox from the mesoporous particles, thus improving the chemotherapeutic of Dox. Compared with CMT or PTT alone, the combination of CMT and PTT can significantly improve the therapeutic efficacy, exhibiting a synergistic effect.

Hydrogen gas was recently discovered to have the potential in the treatment of many diseases [96,97,98], but the current limitation is the low efficacy of stable hydrogen storage and delivery. The Pd atoms are well-known as effective hydrogen carriers and genius catalytic hydrogenation agents [18,99]. To combine the hydrogenation storage/catalysis capacity and photothermal effect as well as the photoacoustic effect of PdNPs, the hydrogenated porphyrin–Pd–organic framework (PdH–MOF) nanoparticles were developed for tumor-targeted photoacoustic imaging (PAI)-guided hydrogenothermal therapy of cancer [45]. PdH–MOF nanoparticles exhibited a high amount of hydrogen loading, sustained release of hydrogen, high tumor-targeting ability, high photothermal effect, and excellent PAI performance. Due to the dual synergetic effect of hydrogen and heating, hydrogenothermal therapy has a low heating requirement in comparison with PTT alone. Pd–MOF nanoparticles displayed a poor PTT efficacy at a low laser power density of 0.5 W/cm^2^; however, PdH–MOF nanoparticles exhibited a significantly higher efficacy of tumor inhibition under the same conditions. The in vitro and in vivo experiment results proved the excellent anti-cancer effects of PdH–MOF.

Besides the two modal effects as discussed above, the multifunctional PdNPs with tri-modal were also developed to enhance the efficiency of cancer treatment. For example, the cancer drug (i.e., Dox) and radiation agent (radioisotope ^131^I) could be loaded to the single porous hollow PdNPs (PHPdNPs) to develop the multifunctional PdNPs with trimodal chemo-, photothermal-, and radiotherapy [44]. With regard to PTT applications, PHPdNPs have excellent photothermal conversion efficiency and excellent photothermal stability in comparison with other mesoporous nanocarriers. The combination of chemo-, photothermal-, and radiotherapy was more effective than a single modal therapy, which was demonstrated by the in vitro test on breast cancer MCF-7 cells. Furthermore, the test on the MCF-7 tumor-bearing mice demonstrated the ability of these nanoparticles on the treatment of cancer with the assistance of a bioimaging diagnosis.

## 11. Toxicity, Pharmacokinetics, and Biodistribution of Palladium Nanoparticles

### 11.1. Toxicity

With the emerging applications of PdNPs on the biomedicine field, the cytotoxicity of PdNPs must be carefully considered to define the suitable strategies for assessing, communicating, and managing of PdNPs to protect the human health and environment. To study the toxic effects caused by exposure to PdNPs, assay tests on in vitro cellular and in vivo animal models are required. 

The cytotoxicity of PdNPs has cell-type selectivity and dose-dependent properties. For example, Wilkinson et al. reported that PdNPs activate the caspase-dependent apoptosis in primary bronchial epithelial cells (PBEC), but not in the lung carcinoma epithelial cell line (A549) [100]. More than 90% of PBEC died after incubation with 25 µg/mL PdNPs for 24 h, but only 10% of A539 cells died under the same condition [100]. As mentioned in Section 6, PdNPs are toxic to cancer cells; thus, PdNPs can work as an antitumor drug. However, the mechanism of antitumor effect needs deeper researches; and in vitro as well as in vivo studies on a wide range of cancer cells and tumor animal models need to be conducted.

Concerning in vivo results, the target effect of PdNPs is on the immune, renal, and endocrine systems [101]. Iavicoli et al. studied the potential immunotoxic impact of immunostimulatory and immunosuppressive effects of PdNPs on Wistar rats [102]. The subchronic exposure to increased doses of PdNPs induced a decreasing trend in serum levels in most of the cytokines investigated [102]. The finding showed that PdNPs strongly affected the immune system of treated rats [102]. Chen et al. [65] reported the size-dependent toxicity of Pd nanosheets. The results revealed that larger-sized Pd nanosheets have more interaction with cellular components (i.e., actin cytoskeleton and fibers) and a higher effect on biological processes (i.e., actin filament-based processes, actin cytoskeleton organization, and myofibril assembly) [65].

### 11.2. Pharmacokinetics

Until now, the pharmacokinetics of PdNPs has not been deeply studied yet; there is a need for pharmacokinetics data to achieve a sufficient understanding of how the body interacts with PdNPs. To study the pharmacokinetics, the adsorption, distribution, metabolism, and excretion of PdNPs need to be considered. Some groups reported the blood circulation half-life of PdNPs. Chet et al. reported that the blood circulation half-life of Pd nanosheet strongly depended on the size of nanoparticles [65]. The different-sized PEGylated Pd nanosheets were intravenously injected into the mice with a dose of 10 mg/kg body weight. The blood was drawn from the tail veins at different time points to build the blood circulation curves. The blood circulation half-life was 32.0 h, 20.0 h, 4.5 h, and 1.7 h for 5 nm, 13 nm, and 80 nm Pd nanosheets, respectively. These results indicated that the larger PEGylated Pd nanosheets were cleared from the blood faster than the smaller PEGylated Pd nanosheets. Tang et al. demonstrated that the smaller size and the modification by glutathione (GSH) were both helpful to prolong the circulation half-life of Pd nanosheets in blood from 0.08 h to 1.25 h [40]. Interestingly, the GSH-modified Pd nanosheets with a size < 10 nm can be cleared easily from the body through the renal excretion route and into the urine.

### 11.3. Biodistribution

The biodistribution of PdNPs depends on their size and the targeting ligand on their surface [13,65]. The biodistribution of Pd nanosheets with a range of sizes was studied by Chen et al. [65]. They found that the accumulation of larger-sized Pd nanosheets in the liver and spleen of the mice was higher than that of smaller-sized Pd nanosheets, even after 30 days. A similar amount of Pd nanosheet accumulated in the heart and lung and that amount was significantly lower than that found in the liver or spleen. After 30 days, Pd nanosheets were almost removed out of the heart and lungs. The smaller-sized Pd nanosheets accumulated in the tumor in a higher number in comparison with larger-sized Pd nanosheets. Bharathiraja et al. proved that Pd@COS with the targeting ligand RGD has a high accumulation in the tumor site in comparison with only Pd@COS [13]. Or, Tang et al. reported that glutathione-modified Pd nanosheets enhanced tumor accumulation in comparison with polyvinylpyrrolidone-capped Pd nanosheets [40].

## 12. Concluding Remarks and Future Outlook

The potential therapeutic properties of PdNPs have been discovered recently; their explored biomedical applications include PTT, PAI, prodrug activities, anticancer activities, antibacterial activities, gene/drug delivery, and biosensors. Some reported studies demonstrated the ability of PdNPs to perform at a similar or even better level than other metal nanoparticles for the same applications in some cases. 

With their unique and genius properties, the new biomedical applications of PdNPs surely will be discovered. For example, PdNPs might be used as probes or diagnostics applications owing to their unique optical properties. PdNPs have a promising potential for the development of highly advanced imaging-guiding therapy, surgery, and drug delivery. The combination of multi-modals into a single system to enhance the effectiveness of therapeutics and reduce the intrinsic side effects might be a trend of further medicine. The remarkable multiple facets and highly porous PdNPs might provide a simple system for combining the effects of multimodal therapy. The novel nanostructures might be synthesized by a combination of the PdNPs and other metals with different ratios for specific biomedical applications.

Moreover, future research needs to be carried out to better understand the antitumor/antibacterial mechanisms of PdNPs at the molecular level and metabolic pathways in in vitro and in vivo evaluations. More studies need to be systematically conducted before PdNPs can be developed into a theranostic agent as an imaging-guided therapy for cancer diagnosis and therapies. Comparison studies between PdNPs and other metal nanoparticles, as well as among PdNPs from different synthesized methods and among PdNPs with different sizes and shapes, are crucial to choose the suitable PdNPs for desired applications. Extensive animal studies and clinical trials are suggested for future researches.

Recently, some possible toxic effects of PdNPs on living systems and their surrounding environment have been reported [101]. However, an understanding of the toxicity of PdNPs is still incomplete and needs more intensive investigations. In addition, some preliminary knowhow on the pharmacokinetics of PdNPs have been explored. Nonetheless, to obtain a comprehensive understanding of how the body interacts with PdNPs, systematic and intensive investigations on the PdNPs’ pharmacokinetics are still needed.

There is still a long way to go for dealing with a lot of challenges to substantiate the application of PdNPs in pharmacy and clinical medicine, and to ensure the smooth transition of these concepts from research to practice. However, the unique properties of PdNPs pose them as possible future important nanoparticles in the nanomedical field.

## Figures and Tables

**Table 1:** Summary of the size, shape, and potential biomedical applications of reported PdNPs.

Pd-Based Materials	Surface Modification	Size	Shape	Potential Biomedical Applications	Ref.
Pd nanosheets		28, 46 and 60 nm	Ultrathin hexagon	Photothermal therapy	[39]
Pd nanosheets	Reduced glutathione	<10 nm	Hexagon	Photothermal therapy	[40]
PdNPs	Methoxy-terminated PEG–thiol	≤30 nm	Porous architecture	Photothermal therapy	[24]
PdNPs	Cancer cell targeting RGD peptide	22.26 ± 0.97 nm	Porous architecture	Photothermal therapy	[13]
				Photoacoustic imaging	
PdNPs embedded in chitosan/polyvinyl alcohol membrane		30.2 ± 17.2 nm	Flower-like shape	Photothermal therapy	[41]
				Wound healing	
Pd nanosheets		16 nm	Hexagon	Photoacoustic imaging	[26]
Pd@gold nanoplates	Thiol-polyethylene glycol	Diameter: 30 nm	Hexagon	Photoacoustic imaging	[42]
		Thickness: 4 nm		Computed tomography	
Pd nanosheets covered hollow mesoporous silica nanoparticles	3-aminopropyltrimethoxysilane	170 nm	sphere	Photothermal therapy	[43]
				Drug delivery	
PdNPs (carrying cancer drug and radiation agent)		58 ± 4 nm	Porous hollow nanoplatforms	Photothermal therapy	[44]
				Drug delivery	
				Radiotherapy	
Hydrogenated porphyrin-Pd-organic framework nanoparticles		93 nm	Framework architecture	Photoacoustic imaging	[45]
				Hydrogenothermal therapy	
PdNPs		2.0 ± 0.1 nm, 2.5 ± 0.2 nm, and 3.1 ± 0.2 nm.	Sphere	Antibacterial therapy	[33]
Pd nanocrystals		~10 nm	Cube Octahedron	Antibacterial therapy	[34]
PdNPs		98 ± 36 nm	Sphere	Antibacterial therapy	[46]
Pd@White tea NPs (synthesized by *Camellia sinensis* leaf extract)		6 nm to 18 nm	Sphere	Antibacterial therapy	[31]
				Anticancer therapy	
PdNPs (synthesized by *Melia azedarach* leaf extract)		10 nm to 20 nm	Sphere	Antibacterial therapy	[47]
				Larvicidal activities	
Pd NPs supported on mesoporous silica (SBA-15–Pd and MSU-2–Pd)		29 ± 9 nm for SBA-15–Pd	Sphere	Anticancer therapy	[48]
		28 ± 5 nm for MSU-2–Pd			
PdNPs (synthesized by *Bauhinia variegata* bark extract)		2.87 nm	Cube	Anticancer therapy	[49]
PdNPs (synthesized by biomass waste petal of *Moringa oleifera*)		10 nm to 50 nm	Sphere	Anticancer therapy	[32]
PdNPs		80 nm to 100 nm	Porous architecture	Gene delivery	[28]
PdNPs (synthesized by chaga mushroom *(Inonotus obliquus)* extract)		~100 nm	Porous architecture	Photothermal therapy	[27]
				Anticancer therapy	
				Drug delivery	
PdNPs	PEG-hydrazide	17 ± 2 nm	Sphere	Drug delivery	[50]
Heterogeneous Pd^0^ resin		~150 µm	Sphere	Prodrug activation (5-fluorouracil and gemcitabine)	[29,30]
PdNPs into self-supporting nanoporous gold wire		10 nm	Nanowires	Biosensors (Dopamine detection)	[51]
